# Field-based assessment of the mechanism of maize yield enhancement by *Azospirillum lipoferum* CRT1

**DOI:** 10.1038/s41598-017-07929-8

**Published:** 2017-08-07

**Authors:** Camille Rozier, Jihane Hamzaoui, Damien Lemoine, Sonia Czarnes, Laurent Legendre

**Affiliations:** 1 Université de Lyon, F-69622, Lyon, France; Université Lyon 1, Villeurbanne, France; CNRS, UMR 5557, Ecologie Microbienne, INRA, UMR 1418 Villeurbanne, France; 2 Université de Lyon, F-69622, Lyon, France; Université Lyon 1, CNRS, UMR 5023 - LEHNA, Laboratoire d’Ecologie des Hydrosystèmes Naturels et Anthropisés, Villeurbanne, France

## Abstract

Plant Growth-Promoting Bacteria (PGPB) of the genus *Azospirillum* are known to enhance root growth and yield in many plant species including cereals. To probe the underlying mechanisms, correlations between modifications of yield and 6-leaf plantlet characteristics were estimated on maize in four fields with contrasting soil properties over two consecutive years using the commercial isolate *A*. *lipoferum* CRT1. In both years, plantlet metabolome, photosynthetic potential and organ morphology were found to display field- and inoculation-specific signatures. Metabolomic analyses revealed that *A*. *lipoferum CRT1* mostly affected sugar metabolism with no suggested impact on N and P assimilation. Mineral nitrogen feeding increased yield but did not affect yield enhancement by the bacterial partner. However, greater improvements of leaf photosynthetic potential correlated with yield diminutions and larger plantlets in all of their proportions correlated with yield enhancements. Bacterial inoculation restored proper seed-to-adult plant ratio when it accidentally dropped below 80%. Only in these cases did it raise yield. All in all, securing mature plant density is hypothesized as being the primary driver of *A*. *lipoferum* CRT1-mediated yield enhancement in maize fields.

## Introduction

Over the past decades, conventional agriculture has become highly dependent on mineral nitrogen, phosphorus and potassium fertilization to increase production and meet world demand^1^. However, these mineral nutrients originate from limited resources and mineral nitrogen lixiviation into fresh water systems is a serious hazard^2^. Despite improvements in plant selection, soil structure and nutrient formulations, current levels of mineral fertilization remain unsustainable^3–5^.

Since their early description in 1978 by Kloepper and Schroth^6^, Plant Growth-Promoting Bacteria (PGPB) represent an attractive source of technologies to maintain yields while reducing mineral nutrition with additional benefits in plant protection against pests and abiotic stresses^7–10^. The majority of PGPB comes from the Firmicutes and Proteobacteria. They engage in associative symbioses on plant root surfaces, or as root endophytes, without the development of specialized structures such as nodules^11–13^. Among PGPB, members of the genus *Azospirillum* have attracted particular attention since they are innocuous and are able to increase yields of many crop species including cereals^14^. A meta-analysis of field trials conducted between 1981 and 2008 notably revealed average enhancements of grain and forage yields of 15 and 27% (n = 91) after wheat inoculation with *Azospirillum* in the absence of nitrogen fertilization^15^. Nevertheless, despite some usage of *Azospirillum*-based commercial products in Latin America^16^, wider usage has been impaired by the variability of the effects as revealed, for example, by the comparative analysis of shoot biomass and grain yield increases in *A*. *brasilense*-inoculated wheat at 297 locations from 2002 to 2006^17^. A review of field assays conducted worldwide^18^ found that all reports of assays conducted in Brazil and Mexico described positive yield-enhancements by *Azospirillum*. However, up to as low as 60% of field assays conducted in Egypt, India and Uruguay concluded to yield-enhancements while only 5 such cases were seen out of 6 years of experimentations in 12 locations in France. Positive effects on yield were even considered as ‘erratic’ in field assays conducted in the USA where no correlation was found with host plant, soil type and N fertilization.

Functional and full genome sequencing studies have attempted to unveil the molecular basis for plant growth-promotion by *Azospirillum*. They have led to the elaboration of several elegant concepts that involve direct or indirect improvements of plant nutrition by *Azospirillum* (reviewed in refs 19 and 20). Several isolates indeed have the genetic ability to transform nitrogen gas into the water-soluble and plant-absorbable form, ammonium^21^, solubilize soil phosphorus^22^ and chelate ferric iron with siderophores^23^. Inoculation of maize seeds with *A*. *lipoferum* leads to a reduction of ascending sap glucose content to lift its feedback down-regulatory inhibition of leaf photosynthetic potential^24^ while wheat plants inoculated with *A*. *brasilense* accumulate higher quantities of photosynthetic pigments^25^. Both activities foster C fixation to sustain increased plant growth. All analyzed *Azospirillum* strains have the potential to alter the hormonal balance of their host by secreting plant hormones such as auxins and cytokinins and by expressing enzymes capable of metabolizing the precursor of ethylene^15, 26^. In support of these concepts, maize plantlets artificially-inoculated with *A*. *lipoferum* display an altered root system phenotype with more numerous, longer lateral roots under field and greenhouse conditions^27^. This allows roots to explore a larger volume of soil in search for nutrients and water. However, the contribution of enhanced mineral nutrient access (especially nitrogen-based) to the final crop yield in a field context is currently a subject of debate^28^ and the potential contributions of *Azospirillum*-induced modifications of photosynthesis and root system architecture to crop yield have also not yet been probed. Because an exogenously added *Azospirillum* strain will only remain on its host root system for up to 57 days^29^, it is hypothesized that early impacts of these bacteria on one, or several, physiological parameters of the host plant such as on root or shoot morphology, leaf photosynthesis or organ metabolite contents (as a result of improved nutrient access for example) are key determinants of bacterial impact on late yields.

In this study, maize seeds were coated with *A*. *lipoferum* CRT1, a commercial *Azospirillum* strain that was originally isolated from the maize rhizosphere^30^ and found to stimulate maize yield and plantlet growth under field^29^ and greenhouse^27, 31^ conditions. Inoculated seeds were sown in 4 different agronomic sites that were close geographically but differed in their soil qualities or agronomic practices. Early responses to *Azospirillum* inoculation on seedling morphology, metabolite content, and photosynthetic potential were measured during two successive years. These were compared to late-occurring measures of yield, such as plant density and grain yield at harvest.

## Materials and Methods

### Bacterial culture and coating on seeds

Organic grade seeds of the maize (*Zea mays* subsp. *mays* L.) genotype Seiddi (Caussade Semences, Caussade, France) were coated by Lesaffre (Agrauxine branch, Beaucouzé, France) with a peat slurry containing *A*. *lipoferum* CRT1 according to previous protocols^29^. Basically, single colonies of pure plate cultures were grown in liquid culture until they accumulated 2–4 poly-*β*-hydroxybutyrate (PHB) bodies. These can be seen under the light microscope and their chemical composition was confirmed by staining with Nil red^32^. These bodies provide food to the bacteria until roots have emerged and can supply exudates. They also help the bacteria stand adverse conditions such as drought and oxidative stress while on the seeds^33^. As bacteria reach this physiological state, proper mobility was checked microscopically and the absence of contaminants determined by plating on Congo red medium according to^34^. The adequacy of the bacterial agent was also assessed by PCR according to^24^. In total, 10^12^ bacteria were harvested by centrifugation (4 000 g, 10 min) resuspended in 200 mL of PCB^+^ medium (tryptone 10 g/L, Yeast extract 5 g/L, glucose 20 g/L, pH 7.0), 400 g dry peat (LegumeFix; Legume Technology Ltd, Epperstone, UK) and wetted with 778 mL UP water to yield a bacterial density of 9.7*10^7^ CFU/mL peat slurry. Two mL of bacterial peat slurry were mixed with 50 g of seeds. Since the average seed weight was 0.37 g, it yielded a final bacterial density of 1.4*10^6^ CFU/seed. A parallel batch of seeds was similarly inoculated at a final density of 10^7^ CFU/seed by starting with 10 times more bacteria. Finally, equivalent lots of seeds were similarly mock-coated with a peat slurry that did not contain the bacterium. Each year, a single batch of an appropriate quantity of seeds sufficient for all field trials was coated, dried in ambient air for a few minutes and stored in the dark at room temperature in paper bags for ca. 3 days until sowing. For germination assays in growth chambers, inoculated seeds were used immediately. In a previous study conducted in the same geographic region with the same coating procedure^29^, the presence of the bacteria was attested on the plant roots, and its tightly adhering soil, after seed germination. Bacteria viability and density were shown to be unchanged for 30 days post-sowing to then sharply decline and be negligible 57 days post-sowing.

### Two days old seedling morphology

Maize seeds that had similar mass (between 0.3609 g and 0.3780 g) and shape were selected in order to reduce germination time heterogeneity. They were inoculated with the bacteria at a final density of 1.4*10^6^ CFU/mL or mock-inoculated as described in the above section on ‘Bacterial culture and coating on seeds’. Seeds were laid on a damp filter paper within closed Petri dishes. Bacteria-inoculated and mock-inoculated seeds were placed in separate Petri dishes. Petri dishes were kept in the dark for 48 h at room temperature before being photographed and the length of the radicles measured.

### Field characteristics and sowing

Commercial fields run according to local agronomic practices were selected because of their geographic proximity (within a 20 km distance) and their differing soil characteristics. One was a fluvic cambisol (called FC - Sérézin-de-la-Tour, France), one was a luvisol (called L - Chatonnay, France) and one was a calcisol (called C - Saint Savin, France). A field neighboring field C (called Corg) and run under organic practices was also selected. The main characteristics of the surface soil (top 5–30 cm) were measured by CESAR Laboratory (Ceyzériat, France) and are summarized in Table 1. All fields had a crop rotation of wheat the year that preceded the assays of this study.

**Table 1:** Field characteristics of the top (5–30 cm) soil layer.

Field	Soil type	GPS coordinates	Altitude (m)	Texture (%)			pH		CaCO_3_ (g.Kg^−1^)	C_organic_ (g.Kg^−1^)	N_total_ (g.Kg^−1^)	C/N	Phosphorus (g.Kg^−1^)	Cation exchange (cmol.Kg^−1^)			
				sand	loam	clay	H_2_O	KCl						CEC	Ca^2+^	Mg^2+^	K^+^
FC	Fluvic Cambisol	N45°28′42.25″ E05°14′53.24″	279	26.9	38.3	34.7	7.1	6.3	4	31.6	3.4	8	0.153	22.8	10.6	0.34	0.38
L	Luvisol	N45°34′01.11″ E05°16′32.62″	535	42.9	42.9	14.2	7.3	6.7	2	21.5	1.6	12	0.171	9.3	5.25	0.17	0.43
C	Calcisol	N45°37′46.77″ E05°16′32.62″	217	15.6	74.1	10.3	8.2	7.7	840	25.9	3.1	8	0.132	9.7	18.05	0.12	0.29
Corg	Calcisol	N45°38′20.82″ E05°16′22.00″	215	27.4	62.5	10.2	8.4	7.9	773	20	2.1	9	0.134	6.5	21.30	0.22	0.31

CEC: cation exchange capacity.

Flat sections of the fields were divided into 5 replicate blocs that were subdivided into 10 (FC), 8 (L and C) or 4 (Corg) plots of 115 m^2^ (FC, C and Corg) or 77 m^2^ (L) according to a split-plot design. Treatments were applied in plots once per bloc (i.e. n = 5 for each treatment in all field) and were randomized in a different manner within each bloc. Fields were sown 3–4 cm below ground at a similar soil temperature on 04/18/2014 (FC), 04/23/2014 (L and C), 04/30/2015 (C, Corg) and on 05/11/2015 (FC and L). Based on soil characteristics, they were sown at densities of 98 000 seeds/ha (FC in 2014 and 2015), 90 000 seeds/ha (C in 2014), 94 000 (C in 2015), 89 000 seeds/ha (L in 2014 and 2015) and 94 000 seeds/ha (Corg in 2015) with an 80 cm spacing between each row (12 rows for FC, C and Corg and 8 rows for L).

Fields L and C received a yearly P-K fertilization (in Kg/ha: 30–60, field C in 2014; 57.25–101.83, field L in 2014; 45–60, field L in 2015) at sowing. Field FC received no P-K fertilization during the assays. At sowing, field L was weeded with Adengo (Bayer, Lyon, France) at 1.5 L/ha in 2014 and Elumis (1.2 L/ha; Syngenta, Bâle, Switzerland) and Bavel 4 S (0.4 L/ha; Syngenta, Bâle, Switzerland) in 2015. It was protected against slugs with Extralugec (Phytoeurop, Levallois-Perret, France) at 4 Kg/ha. Field FC was weeded with Laudis (0.3 Kg/ha; Bayer Crop Science, Monheim am Rhein, Germany), Pampa (0.8 L/ha; Belchim Crop Protection, Lissieu, France), Actirob b (0.8 L/ha; Bayer Crop Science, Monheim am Rhein, Germany) and Banvel 4 S (0.3 L/ha; Syngenta, Bâle, Switzerland) in 2014 and Pampa (1 L/ha; Belchim Crop Protection, Lissieu, France), Callisto (0.8 L/ha; Syngenta, Bâle, Switzerland) and Casper (180 g/ha; Syngenta, Bâle, Switzerland) in 2015. It was protected against slugs with Extralugec (Phytoeurop, Levallois-Perret, France) at 9 Kg/ha in 2014 and 6 Kg/ha in 2015. Field C was weeded with Camix (3.7 L/ha; Syngenta, Bâle, Switzerland) and Emblem Flo (0.8 L/ha; Nufarm, Melbourne, Australia) in 2014 and received no treatment against slugs. Individual plots of fields FC, L and C received either no N fertilization or were fertilized with ammonium nitrate (33.5% N) at maximum levels (120 Kg N/ha for FC and C in 2014, 180 Kg N/ha for L in 2014, 100 Kg N/ha for C in 2015, 160 Kg N/ha for L in 2015, 120 Kg N/ha for FC in 2015 based on soil characteristics at sowing) or intermediate levels as indicated in the result section and in the figure legends. N fertilization was added at the 6-leaf stage unless otherwise stated in the result section and in the figure legends. Field Corg was fertilized with meat flour (120 Kg N/ha) on 04/15/2015 and weeded mechanically with no pesticide added.

### Six-leaf stage physiological measurements

All plots were divided into two equal halves, with the divider perpendicular to the rows. In one half, plants of the central rows were randomly chosen for photosynthetic efficiency, leaf and root architectures, and metabolite analyses. These plants were harvested at the 6-leaf stage a few days prior to N fertilization if applicable on 05/14/2014 (FC), 05/16/2014 (L), 05/22/2014 (C), 06/08/2015 (FC and L) and 05/27/2015 (C and Corg). The maximum quantum efficiency of PSII primary photochemical conversion (Fv/Fm with Fv = Fm-F0) and the efficiency of the overall reaction centers of PSII under light (Ft/Fm’) of the youngest mature leaf (forth from the bottom of the plant) were measured according to Rozier *et al*.^24^ on four randomly-selected plants per plot with a portable photosynthesis yield analyzer (Mini-PAM-II, Walz, Germany) equipped with the clip holder 2035-B. Root and shoot system architectures were respectively recorded on four randomly selected plants per plot with a scanner equipped with WinRhizo and WinFolia softwares (Regent Instruments Inc., Quebec, Canada) according to Rozier *et al*.^24^.

Stem diameter was measured with a hand-held digital caliper at the base of the plants (c.a. 5 mm above the seed). Roots and shoots of four randomly selected plants per plot were oven-dried (24 h, 60 °C) and weighted to estimate their dry weight.

Roots and the most recent mature leaf (forth leaf from the seed) of four randomly-selected plants per plot were sampled and dipped in liquid nitrogen at harvest to inactivate enzymatic reactions. They were lyophilized and the soil attached to the roots carefully removed with a soft brush. Cleaned roots and leaves were cut into 1 mm pieces and ball-milled for 8 min at 30 Hz (TissueLyser II, Qiagen, Hilden, Germany). Metabolites were extracted from 7 mg dry powder with a biphasic water/chloroform/methanol solvent system using ribitol (Sigma-Aldrich, St Louis, USA) as a standard according to Rozier *et al*.^24^. Metabolites were silylated, analyzed by GC-MS (Agilent 7890 A and 7000 A, Santa Clara, USA), annotated using MetAlign and TagFinder softwares and their content estimated with MassHunter software (Agilent, Santa Clara, USA) according to Rozier *et al*.^24^.

### Yield measurements

For each plot, central portions (5 m long) of two central rows of the part not used for 6-leaf stage physiological measurements (section ‘Six-leaf stage physiological measurements’ above) were harvested at the end of the growth cycle on 10/08/2014 (FC and C), 10/16/2014 (L), 10/26/2015 (L) and 10/05/2015 (FC, C, Corg). The grains of all cobs were detached mechanically, weighted and oven-dried until constant weight to estimate the harvested grain yield (dry weight). Harvested plants were also counted to generate an estimate of plant density at harvest.

### Statistical analyses

Unlike otherwise stated, non-parametric Wilcoxon (comparison of two means) or ANOVA (comparison of more than two means) mean comparison tests were conducted with significance and high significance thresholds respectively set at p < 0.05 (*) and p < 0.01 (**). Trends at p < 0.1 (‘) were also recorded during metabolite content comparisons. Monovariate mean comparison tests and multivariate Principal Components Analysis (PCA) or Partial Least Squares-Discriminant analysis (PLS-DA) were carried out with the open source software R^35^ with the RVAidememoire, mixOmics and ade4 packages (downloaded on march 2016).

## Results

### Impact of *A*. *lipoferum* CRT1 on grain yield

Under no-nitrogen input, seed coating with the PGPB *A*. *lipoferum* CRT1 affected grain yields in discrete instances in four geographically close fields contrasted in soil quality and other parameters, over two consecutive years (Table 2). Grain yields increased in field FC in 2014 (+7.9%) and in field L in 2015 (+11.2%). They decreased in field C in 2014 (−10.8%) and field FC in 2015 (−10.0%). There was no significant impact on yield in all other instances.

**Table 2:** Maize grain yields (q/ha) after inoculation (I) and no inoculation (NI) of seeds *with A*. *lipoferum* CRT1.

	FC		L		C		Corg	
	NI	I	NI	I	NI	I	NI	I
2014	96.5 ± 3.1^a^	104.1 ± 4,5^b^	89.5 ± 13.9^a^	91.0 ± 8.7^a^	87 ± 8^a^	77.6 ± 6.6^b^	—	—
2015	62.9 ± 8.5^a^	56.6 ± 7,1^b^	75.6 ± 5.3^a^	84.1 ± 6.7^b^	69.0 ± 5.6^a^	79.7 ± 4.2^a^	77.5 ± 4.3^a^	79.8 ± 3.4^a^

Sub-plots received no nitrogen fertilization in the four fields (FC, L, C, Corg) during the two years of the study. Grain yields are expressed in q/ha. Letters a/b above numbers refer to significance classes at p < 0.05 after two-by-two comparisons between PGPR-inoculated and mock-inoculated conditions (n = 5). Non-parametric Wilcoxon tests were run using blocs as a random variable.

**Figure 1:**
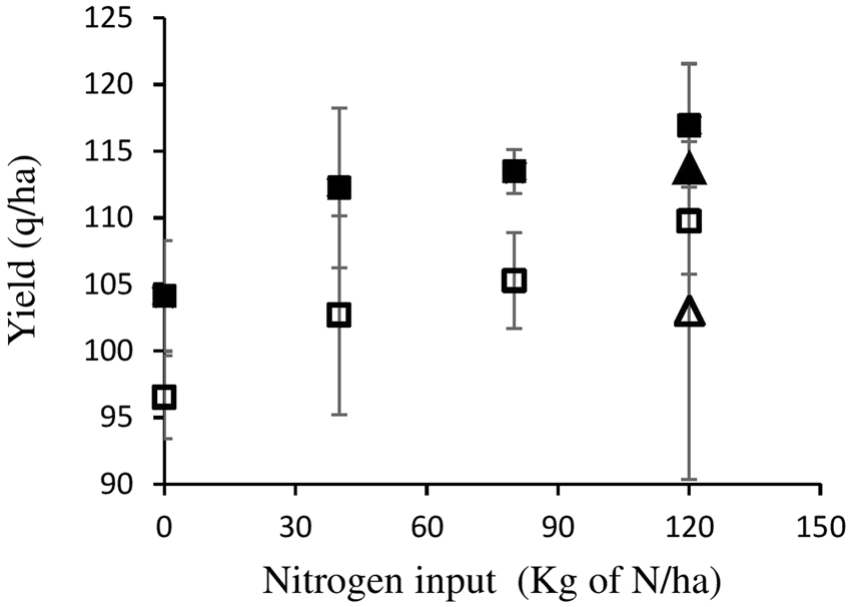
Impact of nitrogen fertilization on maize grain yield. *A*. *lipoferum* CRT1-coated (full symbols) and mock-coated (open symbols) maize seeds were sown in 2014 in field A. Nitrogen was added as ammonium nitrate (33.5% nitrogen N). Nitrogen fertilization was either added once at the 6-leaf stage for all doses (squares) or in two equal amounts at sowing and the 6-leaf stage (triangles) at a total of 120 Kg of N/ha (i.e. 358.2 Kg ammonium nitrate/ha). Means +/− SE are shown (n = 5 blocs in randomized split-plot design).

Excess nitrogen carried over from the previous growing season probably did not cause the lack of yield induction by *A*. *lipoferum* CRT1 in some fields, despite its potential to jeopardize the proper functioning of the interaction between maize and *Azospirillum*. The absence of nitrogen fertilization in some plots of all fields during two consecutive years systematically lowered yields (Table 2) unlike in parallel plots that received full yearly fertilization (data not shown). However, if yield enhancement by *A*. *lipoferum* CRT1 was observed in field L in 2015, unlike in 2014, such an effect was lost in field FC over the same period and never occurred in field C (Table 2). As an additional piece of evidence, serial additions of nitrogen fertilization in field FC in 2014 (40–120 Kg of N/ha) at the 6-leaf stage proportionally increased yield but left the yield-enhancing effect by *A*. *lipoferum* CRT1 unaltered (Fig. 1). Spreading nitrogen input over time (half at sowing and half at the 6-leaf stage) lowered yields slightly compared to a single input at the 6-leaf stage but also did not affect the yield enhancing effect of *A*. *lipoferum* CRT1.

Since yield-enhancement by *Azospirillum* also requires sufficient bacterial coating^36^, a parallel assay was conducted in 2015 in all fields with seeds coated with a 10 times higher bacterial dose (10^7^ CFU/seed instead of 1.4*10^6^ CFU/seed). Not only did such bacterial dose not enhance yields in fields FC, C and Corg, but it suppressed yield enhancement in field L (data not shown) in agreement with previous estimates of the optimum working bacterial dose^36^.

### Impact of *A*. *lipoferum* CRT1 on maize morphology and photosynthetic potential

**Figure 2:**
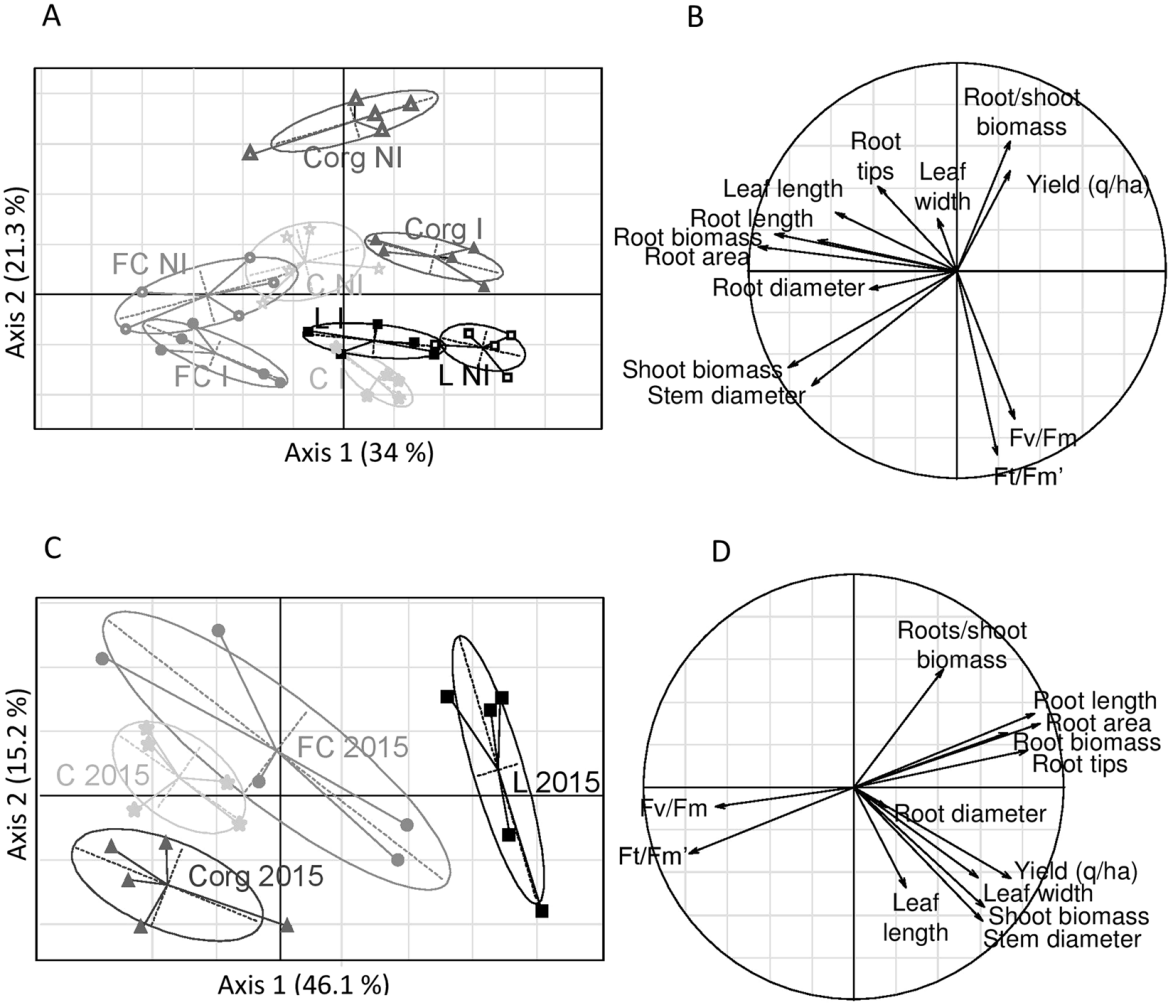
Principal component analysis (PCA) of morphological, photosynthetic and yield characteristics (**A**,**B**) or percent modification of these characteristics by *A*. *lipoferum* CRT1 seed coating within blocs (**C**,**D**). Plants of the 2015 experimental setup (field LC, circles; L, squares; C, stars and Corg, triangles also distinguished by different shades of grey) originating from PGPR-coated (I) or mock-coated (NI) seeds were assessed at the 6-leaf stage except for yield was estimated at the end of the growing season. Four plants were randomly analyzed in each bloc for their morphological and photosynthetic characteristics and the values averaged to yield a single input bloc value for PCA computation. Yields were measured once per bloc. A different shade of grey is given for each experimental condition.

Because *A*. *lipoferum* CRT1 is known to only remain detectable on maize roots up to 57 days post-sowing^29, 31^, ten root and shoot morphological parameters and two photosynthetic potential estimates were recorded at an early seedling stage (6 leaf-stage plantlets) grown under zero-nitrogen fertilization during the 2014 and 2015 field trials for which grain yields are shown in Table 2. A multivariate Principal Component Analysis (PCA) of the 2015 data revealed that mock-inoculated plantlets exhibited a morphological/physiological signature that was unique to each field (Fig. 2A). Organically-grown plants (Corg) were most different in having a higher root-to-shoot biomass ratio and a lower photosynthetic efficiency (Fig. 2B). Plants from field FC differed from the others by having a larger stem diameter and a higher shoot biomass. In all fields, *A*. *lipoferum* CRT1-coated seeds yielded plants with different morphologies than those originating from mock-coated seeds. Their morphological and photosynthetic characteristics remained, nevertheless, field-specific (Fig. 2A,B). Higher yields paralleled higher root-to-shoot biomass ratios, a condition that was most marked in non-inoculated plants of field Corg. Similar conclusions were also drawn with the 2014 dataset (data not shown). A PCA of the plant morphological and physiological characteristics also grouped samples according to their field and inoculation status. Higher yields were also associated with a lower photosynthetic potential and a higher root-to-shoot biomass ratio despite the fact that field Corg was not part of the analysis and that field FC had the highest yield in 2014 unlike in 2015.

To focus on the impact of inoculation on plant morphology, physiology and yield, and to diminish the impact of inter-bloc differences within fields, a similar PCA analysis was conducted on a dataset containing the percentage values of the modification of the plant parameters by *A*. *lipoferum* CRT1 seed coating on each bloc (five blocks per field). It separated the plants of field L from the others on the first axis (Fig. 2C) in parallel with the yield enhancements due to *A*. *lipoferum* CRT1 in this field in 2015 (Table 2). With this representation, modifications of the morphological parameters correlated with each other according to the organ type and negatively correlated with the modifications of the photosynthetic potential (Fig. 2D). Surprisingly, yield increase with *A*. *lipoferum* CRT1 did not associate with a bacterium-induced modification of the root-to-shoot biomass ratio. On the contrary, yield enhancement was associated with a coordinated increase in all of the root and shoot morphological parameters. Inversely, increases of leaf photosynthetic efficiency by *A*. *lipoferum* CRT1 negatively correlated with yield enhancements. Similar conclusions were reached with the 2014 dataset despite plants of field FC (and not L) displaying a yield increase after *A*. *lipoferum* CRT1 seed coating. Plants of this field were indeed separated from the others on the first axis of the PCA plot in 2014 when percent values of the modifications of the plant parameters (and not their absolute values) by *A*. *lipoferum* CRT1 were used for the computation (data not shown). Yield enhancement positively correlated with a coordinated increase of size and weight of all plant parts (roots and shoots) and negatively correlated with a photosynthetic potential increase.

**Figure 3:**
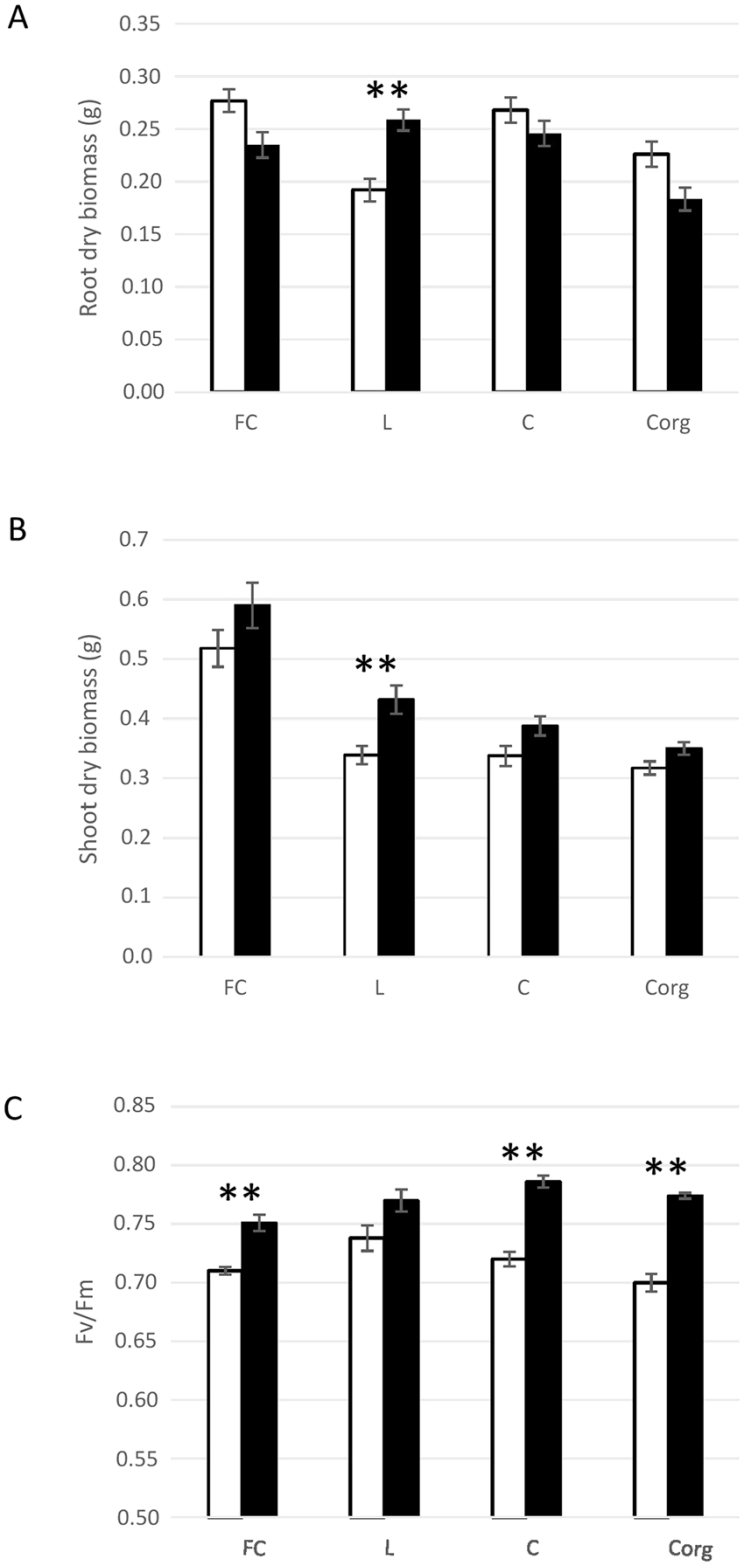
Roots (**A**), shoot (**B**) dry biomass and photosynthesis efficiency (**C**) under differing field and bacterial seed-coating conditions. Measurements were made on mock-inoculated (white bars - NI) or *A*. *lipoferum* CRT1-inoculated (black bars - I) 6-leaf stage plants of all fields (FC, L, C and Corg). Results are expressed as mean ± SE (n = 20). Statistical tests (two-by-two Wilcoxon tests) were conducted to determine significant differences among the 2 conditions (NI and I) and are indicated with stars: *p < 0.05, **p < 0.01.

Plotting the absolute values of single characters of the 2015 dataset confirmed that mock-inoculated plants of field L had the smallest root biomass and were among those that had the smallest shoot biomass. Both parameters gained most from the bacterial inoculation (Fig. 3). Inversely, their photosynthetic potential was already the highest of all mock-inoculated plants and it gained least from the inoculation, which placed plants of all fields at roughly equal values. These values were nearly optimal for maize^37^.

### Impact of *A*. *lipoferum* CRT1 on maize root and leaf metabolite contents

A total of 92 and 82 metabolites were, respectively, annotated in 6-leaf stage maize root and leaf (most recent mature leaf - forth from the bottom of the plant) samples collected in 2015. These included amino acids, carbohydrates, intermediates of the ascorbate or aldarate metabolisms, members of the TCA or urea cycles, polyols, phenolic compounds and purine or pyrimidine metabolites (Supplementary Table S1). Similar sets of metabolites were detected in mock-inoculated and *A*. *lipoferum* CRT1-inoculated plantlets of all fields so that potential differences among treatment groups were quantitative rather than qualitative.

**Figure 4:**
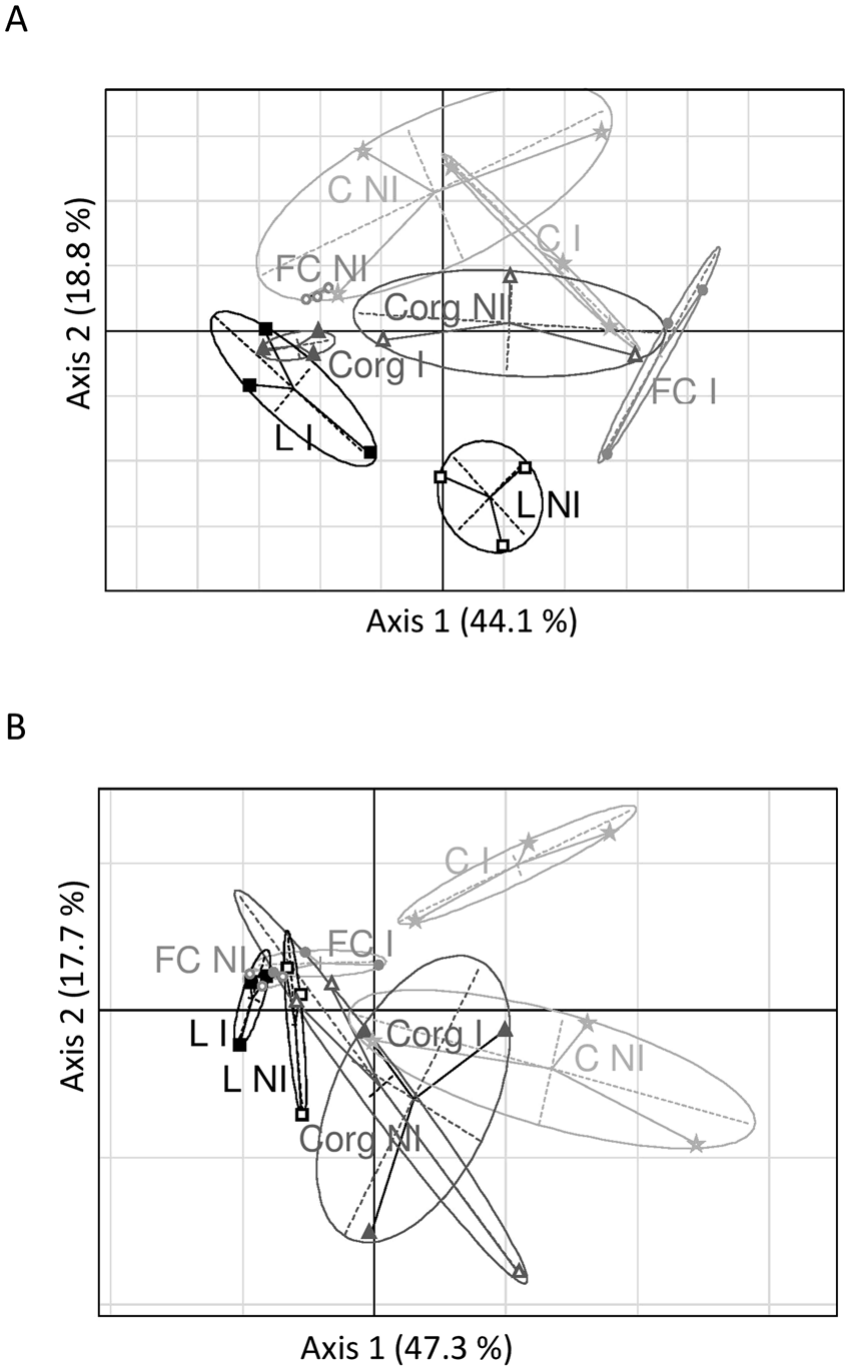
Partial least squares discriminant analysis (PLS-DA) of leaf (**A**) and root (**B**) metabolomes. Analyzed plants originated from the different fields (LC, circles; L, squares; C, stars and Corg, triangles; also distinguished by different shades of grey) and were either mock-inoculated (empty symbols) or inoculated with *A*. *lipoferum* CRT1 (full-colored symbols).

A multivariate analysis of the root and leaf metabolite datasets revealed chemical signatures that were specific to sites and *A*. *lipoferum* CRT1 inoculation (Fig. 4). Differentiation of metabolomes according to treatment conditions was greater in leaves than in roots. Differentiation of leaf metabolomes was nevertheless not total for all conditions (non-inoculated plants of fields FC and C, or inoculated and non-inoculated plants of field C have, for example, overlapping distribution ellipses on Fig. 4A). But plants of these conditions had different root metabolomes (Fig. 4B).

*A*. *lipoferum* CRT1 affected the content of 13 and 28 metabolites in roots and leaves, respectively. Though these belonged to varied metabolite classes, most were carbohydrates or carbohydrate metabolism-related metabolites (Table 3). Not a single metabolite had its content significantly modified (at p < 0.05) by *A*. *lipoferum* CRT1 in the plants of at least two of the four fields (Table 3). Glutamine was the only metabolite which content was significantly affected in both the leaves and roots of the plants of the same field (FC).

**Table 3:** Metabolite content changes in roots (A) or leaves (B) after seed inoculation by *A*. *lipoferum* CRT1.

Organ	Substance name	FC	L	C	Corg
Root	Cadaverine	270.6 ± 1.6*	−20.2 ± 11.6	7.3 ± 6.6	−1.7 ± 9.3
	Cellobiose^#^	127.7 ± 50.1	−63 ± 15.1′	35.8 ± 107.7	9.5 ± 47.9
	4-hydroxy- *cis*-cinnamic acid	145.6 ± 30.0	−52.5 ± 8.1′	NA	−49.31 ± 0.3
	Dihydrocoumarin	39.2 ± 2.7*	−54.9 ± 20.1	NA	NA
	Galactinol^#^	129.2 ± 26.7	−50.2 ± 26.9	−60.1 ± 3.5*	27 ± 1.8
	Glutamine	25.3 ± 1.9*	3.5 ± 6.9	21.1 ± 5.8	−19.5 ± 2.8
	Isomaltose^#^	265.8 ± 6.1*	−76.6 ± 6.0	−22.5 ± 0.7	90 ± 8.1
	Maltitol^#^	81 ± 78.1	−35.3 ± 7.8	−65.8 ± 1.1*	291.6 ± 48.4
	Maltose^#^	176 ± 24.7′	−21.5 ± 24.0	−21.5 ± 1.9	510 ± 69.3
	Phosphoric acid	56.6 ± 17.3	−9.9 ± 1.7	−0.7 ± 1.1	80.9 ± 7.2′
	Ribose-5-phosphate^#^	134.6 ± 25.1	−16 ± 7.5	−58.4 ± 9.7′	26.8 ± 23.4
	Sophorose^#^	198.4 ± 19.8′	14.7 ± 9.8	−32.7 ± 10.4	222 ± 31.2
	A264005	164 ± 78.9	−55.2 ± 13.1′	−10.4 ± 51.8	46.4 ± 20.8
Leaf	*Beta*- alanine	118.0 ± 2.6*	11.6 ± 2.5	91.9 ± 104.0	−73.1 ± 59.1
	Cinnamic acid, trans-	111.2 ± 4.5′	−76.4 ± 4.2′	77.1 ± 7.0	−61.6 ± 3.4′
	Ferulic acid, trans-	NA	−77.5 ± 0.3	100.8 ± 49.8	−66.21 ± 0.2*
	Fructose^#^	−6.7 ± 52.5	88.1 ± 15.9	−32.2 ± 74.5	−61.6 ± 13.8′
	Galactinol^#^	462.7 ± 77.1	NA	117.3 ± 49.0	−69.2 ± 12.9′
	Galactose^#^	36.9 ± 35.5	−51.0 ± 10.7	34.7 ± 24.7	−62.9 ± 7.2*
	Glucose^#^	6.9 ± 73.1	136.3 ± 46.8	−28.8 ± 33.6	−27.1 ± 1.4*
	Glucose-6-phosphate^#^	59.0 ± 34.6	−22.2 ± 0.8	23.5 ± 17.2	−52.7 ± 1.4′
	Glucuronic acid^#^	−266.8 ± 43.8	−47.5 ± 0.1	−3.0 ± 1.9	−71.3 ± 7.2*
	Glutamine	256.6 ± 1.1*	−61.4 ± 2.1′	85.0 ± 3.9	−64.5 ± 31.1
	Gulonic acid^#^	−3.9 ± 0.7	−78.6 ± 16.3	233.4 ± 0.9′	−8.9 ± 3.4
	Gulonic acid, 2-oxo-^#^	23.0 ± 63.5	89.4 ± 6.1*	−24.8 ± 94.7	−26.9 ± 29.1
	Isobutanoic acid, 3-amino-	82.1 ± 0.2′	−57.4 ± 5.5′	77.1 ± 28.9	−77.1 ± 3.6′
	Maltose^#^	29.12 ± 1.3	−11.6 ± 1.3	−29.0 ± 8.2	−24 ± 0.1*
	Mannosamine, N-acetyl-^#^	531.7 ± 6.9*	−62.6 ± 9.6	145.5 ± 16.5	−60.7 ± 0.6′
	Mannose^#^	62.4 ± 269.8	−56.5 ± 82.9	33.0 ± 212.5	−68.3 ± 7.2*
	Quinic acid	61.9 ± 19.7	−58.8 ± 8.5	37.9 ± 15.4	−65.5 ± 3.9*
	Rhamnose^#^	−329.7 ± 52.0	−62.1 ± 75.3	82.6 ± 220.2	−65.8 ± 19.5′
	Ribose^#^	64.7 ± 69.8	−62.9 ± 55.0	−2.4 ± 1.2	50.7 ± 1.4*
	Tagatose^#^	27.1 ± 6.6	11.4 ± 1.3	−8.8 ± 22.4	−66.8 ± 2.5*
	A231002	27.1 ± 128.5	82.9 ± 11.8′	−1.8 ± 221.9	14.0 ± 84.0
	A257011	62.6 ± 37.8	−54.1 ± 52.5	203.5 ± 55.0	−76.8 ± 3.6′
	A115002	268.3 ± 2.3*	−89.3 ± 22.7	126.6 ± 6.1	−40.5 ± 4.9
	A214003	47.6 ± 18.8	−49.7 ± 17.4	9.4 ± 1.3	−54.0 ± 1.1*
	A250008	731.5 ± 21.7	−63.4 ± 31.3	33 ± 18.4	−71.7 ± 9.5′
	A255001	226.7 ± 55.6	−78.3 ± 8.3*	19.8 ± 0.3	−46.1 ± 1.4′
	A268008	105.2 ± 1.5*	−73.4 ± 53.5	111.4 ± 31.5	−84.1 ± 2.1*
	A311002	76.1 ± 7.6′	−65.4 ± 33.4	18.9 ± 59.7	−77.0 ± 58.1

Ratios of mean content values of inoculated over mock-inoculated plantlets are listed and expressed as percentage values +/− percentage relative standard error. Wilcoxon non-parametric tests were conducted for each metabolite to compare inoculated and non-inoculated plants mean values for each field (FC, L, C and Corg). Only substances displaying significant content changes (*p < 0.05; ′p < 0.1) are listed. NA indicates that the metabolite was detected in less than 2 of technical replicate samples. Metabolites were identified by comparison with a standard and are listed in alphabetical order. Unidentified metabolites are named according to their code in the Golm Metabolome Database (http://gmd.mpimpgolm.mpg.de/). Carbohydrates and carbohydrate-related metabolites are marked with the pound sign (^#^).

### Impact of *A*. *lipoferum* CRT1 on two-day maize morphology and seed-to-adult plant ratio

**Figure 5:**
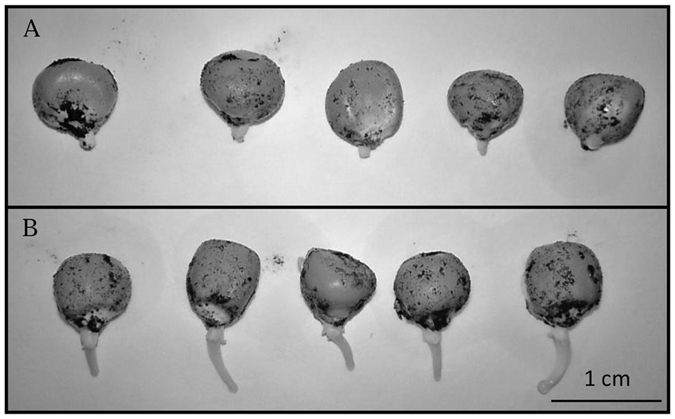
Mock-inoculated (**A**) and *A*. *lipoferum* CRT1-inoculated (**B**) maize seeds 48 h post-inoculation. Five representative seeds of each treatment group (n = 100) have been pictured. The black marks on the seeds are due to the presence of the peat slurry used to inoculate, or mock-inoculate the seeds. A second biological replicate gave similar results.

Because yield enhancement was not linked to a metabolic signature and did not parallel a modification of the root-to-shoot biomass ratio but rather correlated with plants which were larger in all of their proportions, modifications of seed-to-adult plant ratio by the bacterial symbionts were estimated and plant morphology was analyzed as early as two days after sowing (right after germination, i.e., radicle emergence). Under controlled laboratory conditions, germination success was greater than 95% and was not affected by *A*. *lipoferum* CRT1 seed coating (data not shown). However, two days old inoculated seedlings had longer radicles (0.13 ± 0.02 cm and 0.47 ± 0.06 cm, respectively, for mock-inoculated and *A*. *lipoferum* CRT1-inoculated seeds, p < 0.001, Student *t*.test, n = 100 and 2 biological replicates) (Fig. 5). Mock-inoculated seeds had radicles of similar length as untreated ones (0.16 + 0.04 cm, p = 0.43, Student *t*.test, n = 100). Because similar measurements cannot be done under field conditions, plant density was measured at harvest (Table 4). Based on initial sowing density, it could be estimated during the two years of this field study that 80.3–99.4% of the seeds generated adult plants at harvest except in field FC in 2014 and L in 2015 where successes under mock-inoculated conditions were 71.4% and 75.1% respectively. Interestingly, it was only under these conditions that *A*. *lipoferum* CRT1 inoculation significantly raised seed-to-adult plant ratio to values equivalent to those of the other conditions (85.9% and 89.5%, respectively) and increased grain yield (Table 2).

**Table 4:** Percentage of sown seeds making mature plants at harvest.

Year	FC		L		C		Corg	
	NI	I	NI	I	NI	I	NI	I
2014	71.4 ± 5.8^a^	85.9 ± 6.4^b^	80.3 ± 9.4^a^	81.7 ± 3.0^a^	86.3 ± 3.5^a^	81.7 ± 8.2^a^	—	—
2015	92.3 ± 8.0^a^	99.4 ± 5.4^a^	75.1 ± 8.0^a^	89.5 ± 6.2^b^	94.0 ± 3.7^a^	95.4 ± 3.5^a^	85.3 ± 2.6^a^	86.2 ± 3.3^a^

Sub-plots received no nitrogen fertilization in the four fields (FC, L, C, Corg) during the two years of the study. Mature plants at harvest were counted and then expressed as mean percentages (%) of initially sown seeds. Letters a/b below numbers refer to significance classes at p < 0.05 after two-by-two comparisons between *A*. *lipoferum* CRT1-inoculated and mock-inoculated conditions (n = 5). Non-parametric Wilcoxon tests were run using blocs as a random variable.

## Discussion

This large field-based study confirmed that cereal seed coating with *A*. *lipoferum* CRT1 can enhance agronomic yield in fields as already established with this isolate and other *Azospirillum* isolates^15, 38, 39^. This stimulatory effect had been found in several studies to be variable^20, 40^ as in this study. Negative impact on yield was also observed occasionally. Because the experimental setup of this study was re-sown two years in a row in an identical manner, it is further suggested that yield enhancement is dependent on a combination of soil and weather characteristics to occur on different sites on different years. These two parameters being multifactorial, and acting jointly on plants, it is hard, at this stage, to speculate about which combination is most beneficial, or deleterious. But the short survival of *A*. *lipoferum* CRT1 on maize roots (less than 57 days post-sowing) has already led to the suggestion that early events in the plant life are crucial.

Looking at young seedling morphological and physiological characteristics, this study confirmed the growth-promoting activity of *A*. *lipoferum* on the shoots and roots of maize seedlings and its positive impact on leaf photosynthetic potential as already described by many studies mostly conducted under artificial conditions such as in pots and *in vitro* ^30, 31, 36^ including a recent study^24^ making use of the same *A*. *lipoferum* isolate, the same maize cultivar, and the same soil (of field FC) as in this study. Plants of some blocs of several fields notably displayed a modified root system architecture with more numerous and longer lateral roots and a higher root-to-shoot biomass ratio, an early landmark of *Azospirillum* success on plants (reviewed by Bashan *et al*.^41^). Nevertheless, although this study revealed a positive correlation between the root-to-shoot biomass ratio of plantlets and mature plant grain yield, the modification of this parameter by *A*. *lipoferum* CRT1 did not correlate with its ability to enhance yield so that other mechanisms control this phenomenon. Modifications of the proportions of plant parts have been hypothesized to be initiated by auxins produced and secreted by *Azospirillum* ^42, 43^. A full genome sequencing of the *Azospirillum* strain of this study did not reveal the presence of the *ipdC* gene commonly responsible for producing the major plant auxin, IAA, by *Azospirillum* (data not shown). However, the production of other auxin-like substances or the existence of alternative biosynthetic pathways cannot be ruled out.

Enhancing plant nutrition is a more conventional way for raising yield. Adding mineral nitrogen did indeed proportionally raise maize grain yield in this study. However, a metabolomic analysis of the leaves and roots of the plants of this study provided no evidence for any significant modification of N and P assimilation by *A*. *lipoferum* CRT1, in support of several analyses of the impact of N mineralization and P solubilization by *Azospirillum* spp that concluded that these phenomena have, at best, a meager impact on the plant host^28, 44–46^. Indeed, the nitrogen and phosphorus nutrition-related metabolites glutamine^47, 48^ and phosphoric acid were among the metabolites whose contents was modified by *A*. *lipoferum* CRT1 seed coating (Table 3). However, phosphoric acid displayed a trend of content change that was only positive and significant at p < 0.1 (Wilcoxon test) in the plants of field Corg, and glutamine content was not affected in the leaves and roots of plants of fields C and Corg while it displayed a negative change in the leaves of the plants of field L, the only field where yield-enhancement was observed in 2015 when the metabolome analyses were conducted. It rather appeared in this study that the presence of *A*. *lipoferum* CRT1 is perceived by the plant as an additional environmental cue to adjust leaf and root morphologies and metabolomes that were already well differentiated by other field-related environmental stimuli such as weather and soil composition, structure and microbiome (Fig. 4). But neither modifications of the relative proportions of plant parts and/or of their metabolite contents were contributors of yield enhancement by *A*. *lipoferum* CRT1.

Several data of this study suggest that securing adult plant density may be the primary mechanism of yield enhancement by *A*. *lipoferum* CRT1. Germination is a much slower and more uncertain event in fields than in artificial cultures. Seeds, and very young seedlings, are at high risk of dehydration, chilling or predation until their root system is long enough to reach secure soil layers where water content and temperature are more stable. Under the field conditions of this study, the seed-to-adult plant ratio was generally greater than 80%. Only occasionally did it drop to around c.a. 75%. However, it was under these conditions that *A*. *lipoferum* CRT1 raised seed-to-adult plant ratio and grain yield. Proper levels were actually restored. Hypothesizing a link between securing adult plant density and yield enhancement by *A*. *lipoferum* CRT1 is consistent with the observation of this study and of a meta-analysis on yield enhancement by *Azospirillum* species in cereals^15^ where N-addition or deprivation had no impact on yield enhancement by *A*. *lipoferum* CRT1. It is also consistent with the dual impact of soil and weather on yield enhancement by *A*. *lipoferum* CRT1 because both parameters influence seed germination, seedling establishment and pest development. Because no impact of pathogens or predators was observed on 3-leaf or older plants, most mortality occured before, during one of the many sequential steps and biochemical, and gene regulatory, processes that make-up germination and post-germination^49^. It is also during this time that *A*. *lipoferum* CRT1 is present on seed and roots. The data of this study suggest that earlier development of roots two days after sowing leads to more advanced, larger plants (in terms of shoots and roots) at the 6-leaf stage. This faster plant development may bring plantlets increased tolerance to climate havoc. Further investigations will now aim to narrow down the specific step(s) that is (are) improved by the presence of *A*. *lipoferum* CRT1.

Occasionally, *A*. *lipoferum* CRT1 seed coating decreased yield. This phenomenon which received virtually no attention in the literature was not found in this study to correlate with a decreased seed-to-adult plant ratio. But it was found to negatively correlate with modifications of the photosynthetic potential. Plants that had the weakest photosynthetic potential at the 6-leaf stage were most affected. These belonged to fields that had optimum germination success. An independent 3-year survey using a different PGPB species also established a similar correlation^50^. This is surprising because this physiological parameter is used routinely by many agronomists to correct feeding shortages in maize fields at the grain filling stage^51, 52^ and enhancement of host plant photosynthetic potential^24^ and photosynthetic pigments^25^ by *Azospirillum* species is a common observation. Nevertheless, the molecular mechanisms of action of *Azospirillum* species on their host photosystem functioning and consecutive impact on plant growth are unknown and may be more complex than anticipated. Using controlled growth conditions, a recent metabolomic analysis indicated that *A*. *lipoferum* CRT1 lowers glucose content in the upwardly moving xylem sap to lift the down-regulatory effect that this substance exerts on the efficiency of leaf photochemical conversion^24^. More investigations are now required to understand the links between photosynthesis optimization in seedlings, its regulation by *A*. *lipoferum* CRT1, and yield at harvest.

## Electronic supplementary material

Supplementary Information
