# Supplementary material for: Field-based assessment of the mechanism of maize yield enhancement by Azospirillum lipoferum CRT1
Source: Sci Rep. 2017 Aug 7;7:7416. doi: 10.1038/s41598-017-07929-8 (PMC5547117; doi:10.1038/s41598-017-07929-8)
Supplement: Supplementary file 1 — Supplementary Information [file 41598_2017_7929_MOESM1_ESM.doc]

**Field-based assessment of the mechanism of maize yield enhancement by *Azospirillum lipoferum* CRT1**

Camille Rozier^1^ · Jihane Hamzaoui^1^ · Damien Lemoine^2^ · Sonia Czarnes^1^ · Laurent Legendre^1^

^1^ Université de Lyon, F-69622, Lyon, France; Université Lyon 1, Villeurbanne, France; CNRS, UMR5557, Ecologie Microbienne, Villeurbanne, France; INRA, UMR1418, Villeurbanne, France

^2^ Université de Lyon, F-69622, Lyon, France ; Université Lyon 1, Villeurbanne, France ; CNRS, UMR 5023 - LEHNA, Laboratoire d'Ecologie des Hydrosystèmes Naturels et Anthropisés Villeurbanne, France

Supplementary Table S1: Percent variation of metabolite contents in the root (A) and in the shoots (B) of Seiddi maize genotype upon *A. lipoferum* CRT1 inoculation in the four parcels: FC, L, C and Corg.

| ALINK Excel.Sheet.12 "C:\\Users\\camille\\Desktop\\Supplemental S1.xlsx" S1!L1C17:L104C22 \a \f 4 \h \* MERGEFORM |  |  |  |  |  |
| --- | --- | --- | --- | --- | --- |
| Metabolic pathways | | Metabolic variation I vs NI (%) | | | |
|  |  | FC | L | C | Corg |
| SUGAR METABOLISM | |  |  |  |  |
| *Starch + sucrose :* | |  |  |  |  |
|  | Maltose | 119.3±33.8 | -45.5±17.5 | -16.5±27.2 | 28.7±8.4 |
|  | Sucrose | 117.8±75.8 | -43.7±10.5 | 9.9±5.2 | 26.3±34.6 |
|  | Trehalose. alpha.alpha'-. D- | 25.7±14.8 | -19±7.6 | -45.1±13.4 | 216.4±63.2 |
|  | Xylose | 26.5±4.9 | -34.2±9.5 | 15.5±7 | -38.3±9.2 |
| *Fructose + mannose :* | |  |  |  |  |
|  | Sorbose | 40.5±9.8 | -26.3±13.1 | -63.4±47.8 | 11.7±16.1 |
|  | Mannose | 113.7±69.2 | -40.1±12.3 | -37.4±14.5 | -53.5±17.9 |
|  | Rhamnose | NA | 94.8±31.8 | 30.7±16.3 | 73.9±21.1 |
| *Galactose :* | |  |  |  |  |
|  | Galactose | 98.8±41.1 | -40.1±30.9 | -65.8±39.3 | -66.4±46.8 |
|  | Tagatose | 74.3±13.8 | -52.8±13.8 | NA | 54.7±38.4 |
| *Pentose phosphate :* | |  |  |  |  |
|  | Ribose | -21.2±4 | -40.5±11.5 | -15.2±17.3 | -10.8±1.1 |
|  | Gluconic acid. 2-oxo- | -17.3±8 | -49.5±16.1 | -51.1±17.6 | -13±24.8 |
|  | Glyceraldehyde | 121.5±71.2 | 2.5±0.6 | -23.5±6.9 | -59.5±15.8 |
|  | Ribulose | 111.6±51.1 | -23.7±14.5 | 3.1±1.8 | -18.5±5.3 |
| *Glycolysis + neoglucogenesis :* | |  |  |  |  |
|  | Glucose | 126.8±45.3 | -44±18.2 | -24±17.9 | -32.7±16.2 |
|  | Glucose-6-phosphate | -36.3±10.1 | -46.9±12.8 | -72.7±25.6 | -43±15.6 |
|  | Phosphoenolpyruvic acid | 26.8±9.4 | -83.8±7.7 | -35.7±16.1 | 1.8±1.3 |
|  | Galactose-6-phosphate | 221.9±81.7 | -40.2±24.1 | -17.6±8.2 | -43.5±15.9 |
| ASCORBATE AND ALDARATE METABOLISM | |  |  |  |  |
|  | Glucuronic acid | 60±13.4 | -60±12.9 | -27.2±10.8 | -58.9±12.7 |
|  | Threonic acid | NA | -92.1±74.7 | NA | -9.4±2.1 |
|  | Galacturonic acid | NA | -46.3±12.1 | -26.5±63.4 | -75±24 |
| TCA CYCLE | |  |  |  |  |
|  | Citric acid | 119.9±57.6 | -37.1±15 | -33.5±20.1 | -47.6±30.2 |
|  | Malic acid | 61.7±6.2 | 132.9±35.4 | NA | 38.4±9.5 |
|  | Citric acid. 2-methyl- | 19±5 | -74.1±25.8 | -27.6±10.3 | 109.8±46.3 |
|  | Itaconic acid | 377.9±182.9 | NA | NA | NA |
|  | Aconitic acid. cis- | 118.8±41.1 | -12.8±7.2 | -48.8±16.3 | NA |
|  | Glutaric acid. 2-oxo- | 45.6±13.1 | 383.7±76.8 | NA | 15.5±2.2 |
| AMINO ACIDS METABOLISM | |  |  |  |  |
| *Lysine :* | |  |  |  |  |
|  | Lysine | 41.7±10.9 | -57.4±7.2 | -50.8±9.9 | -46.4±14.9 |
| *Alanine, aspartic acid, glutamate :* | |  |  |  |  |
|  | Glutamic acid | 111.3±36.8 | 25.9±7.1 | -29.7±8.1 | 259.5±68.3 |
|  | Asparagine | 107.6±72.7 | -8.9±15.9 | -60.7±8 | 232.1±36.7 |
|  | Aspartic acid | 147±81.7 | 15±12.8 | 63.7±17.3 | 83.8±41.4 |
| *Cysteine, methionine :* | |  |  |  |  |
|  | Methionine | 54.3±24.6 | -50.3±4.7 | -70.5±9.5 | 12.9±13.5 |
| *Arginine, proline :* | |  |  |  |  |
|  | Proline | 113±40 | NA | 15.8±9.8 | 46.3±22.1 |
| *Glycine, serine, threonine :* | |  |  |  |  |
|  | Glycine | -3.3±1 | 14.5±8.1 | -71.3±20.2 | -67.9±17.8 |
|  | Serine. O-acetyl- | NA | NA | -33.3±11.7 | 195.1±91.3 |
|  | Homoserine | -27.4±7 | -17.8±3.5 | -29.6±8.3 | 564.3±94.8 |
|  | Threonine, L- | 120.1±38.2 | -39.9±12.8 | -35.8±18.7 | -4.1±1.4 |
|  | Serine | 58.7±23.1 | 1±6.2 | -37.9±14.5 | 7.6±8.1 |
| *Valine, leucine, isoleucine :* | |  |  |  |  |
|  | Isoleucine | -47.7±18.5 | -30.4±15.4 | -85.3±56.7 | 34.3±12.2 |
|  | Leucine | 78.1±14.6 | 27.9±12.4 | 36.8±8 | 25.4±3.4 |
| *Histidine :* | |  |  |  |  |
|  | Histidine | 116.1±47.9 | -75.6±11.9 | 32.5±8.2 | 90.8±29.5 |
| *Aromatic amino acids :* | |  |  |  |  |
|  | Shikimic acid | 10.6±5.8 | -49.9±14.9 | 13.6±4.1 | -10.9±7.5 |
| UREA CYCLE METABOLISM | |  |  |  |  |
|  | Citrulline | -59±11.4 | NA | -41.6±10.5 | 8.9±1 |
|  | Ornithine-1.5-lactam | 27.5±14.2 | -28.3±6.3 | NA | -40.9±11.4 |
| POLYOLS | |  |  |  |  |
|  | Mannitol | 46.8±18.7 | -55.4±12.3 | -27.1±16.9 | -14.7±7.3 |
|  | Inositol. myo- | 42.1±10.9 | -28.6±13.5 | -22.5±8 | 17±8.9 |
|  | Xylitol | -12.6±4.6 | -13.1±5.2 | -33.5±29.3 | -5.9±2.4 |
| PHENOLIC COMPOUNDS | |  |  |  |  |
|  | Ferulic acid. cis- | 714.6±85 | 237.9±284.2 | -55±27.1 | -61.8±26.3 |
|  | Ferulic acid. trans- | 213.9±90 | NA | -29±7 | NA |
|  | Caffeic acid. cis- | 0.5±35.2 | -25.4±22.5 | -54.9±20.9 | -64.2±50.2 |
|  | Cinnamic acid. 4-hydroxy-. trans- | 125.6±51.1 | -23.1±8.8 | -38.9±9.4 | -58.2±12.2 |
| PURINE AND PYRIMIDINE METABOLISM | |  |  |  |  |
|  | Uracil | 127±19 | -53.9±1.1 | -52.9±23.3 | 68.3±15 |
|  | Thymine | 93.2±28.4 | 21.4±8.4 | -76.3±16.6 | -65.4±13 |
|  | Thymidine | -31.5±19.9 | -14.3±9.2 | -12.7±5.1 | 169.4±35 |
|  | Inosine | 220.3±62.1 | -18.7±3.8 | -22.4±34.4 | 33.3±20.5 |
| OTHER PATHWAY METABOLISM | |  |  |  |  |
|  | Ethanolamine | NA | NA | 79.6±18.3 | NA |
|  | Triethanolamine | 1.9±1.6 | -5.2±9 | -41.8±21 | 101±46.6 |
|  | Glucose. 2-amino-2-deoxy- | 65.3±14 | -64±12.1 | -44.8±28.5 | -35±NA |
|  | Glutaric acid | 415±43.8 | 57±27.4 | NA | NA |
|  | Erythrose | 218.5±83.7 | 21.4±9.2 | -50.3±27.1 | -56.4±5.9 |
|  | Ribonic acid | 39.4±10.2 | -41.6±13.4 | 4.5±1.9 | -0.1±0.3 |
|  | Galactosamine. N-acetyl- | NA | NA | -26.7±10.2 | 86.6±32.1 |
|  | Glucopyranose. D- | 105.5±40.2 | 3.1±0.3 | -53.6±41.3 | -61±22.5 |
| UNKNOWN COMPOUNDS | |  |  |  |  |
|  | similar to Glycerolaldopyranosid | 9.7±3.7 | NA | 21.9±7.3 | 64.5±16.1 |
|  | similar to Diethanolamine | NA | NA | 347.8±63.8 | NA |
|  | similar to Fructose Derivate | 86.3±40.1 | NA | NA | -71.9±20.9 |
|  | A292006 | 75.3±19.8 | NA | 0.1±2.3 | 26.7±8.1 |
|  | A304001 | -61±13.6 | -34.1±10.5 | -9.8±3.3 | 285±81.1 |
|  | A269003 | 121.8±26.8 | -47.4±17.1 | -32.4±37.4 | 18.6±45 |
|  | A254009 | NA | NA | -15.1±12.1 | 4.1±1.9 |
|  | A259001 | 517.3±100.6 | -51.6±10.1 | 49.8±20.1 | -16.4±6.8 |
|  | A218003 | NA | -55.8±25.9 | -40.8±16.2 | -59.6±19.1 |
|  | A176026 | -12.3±5.6 | -1.3±0.4 | 73.8±20.4 | -25.8±9.8 |
|  | A176025 | -40.5±15.1 | -56.8±19.5 | 196.2±46.8 | -36±24.8 |
|  | A170017 | 21.6±9.4 | 4.8±2.1 | -13.9±4.6 | -11.8±4.4 |
|  | A166002 | 58.5±15 | -24.2±13.3 | -54.7±8.4 | 219.6±84.3 |
|  | A166016 | 70.3±28.2 | -72±11 | -13.1±7.5 | 7.1±2.7 |
|  | A171016 | NA | -81.9±20 | -58.4±21.5 | 7.7±2.8 |
|  | A237007 | 83.1±13.4 | -21.1±7.5 | -44.4±48.7 | -30±11.8 |

| BLINK Excel.Sheet.12 "C:\\Users\\camille\\Desktop\\Supplemental S1.xlsx" S1!L111C1:L187C6 \a \f 4 \h \* MERGEFORMAT  Metabolic pathways | | Metabolic variation I vs NI (%) | | | |
| --- | --- | --- | --- | --- | --- |
|  |  | FC | L | C | Corg |
| SUGAR METABOLISM | |  |  |  |  |
| *Sugar dimers :* | |  |  |  |  |
|  | Sophorose | -17.9±8.2 | NA | -24.5±5.4 | NA |
|  | Gentiobiose | 441.3±30.8 | -32.2±3 | 59.3±19.7 | -73.6±25.7 |
| *Starch + sucrose :* | |  |  |  |  |
|  | Isomaltose | -4.1±193.5 | 15.6±6.9 | -23.5±34.6 | -53.3±18.8 |
|  | Sucrose | 85.3±33.8 | 40.1±13.7 | -32.5±26.1 | 5.5±3.2 |
|  | Trehalose, alpha,alpha'- D | 77.4±46.4 | 15.7±6 | -24±8.1 | -0.9±11 |
| *Fructose + mannose :* | |  |  |  |  |
|  | Mannose | 3.4±4.7 | 105.9±30.2 | -30.8±12.1 | -21.5±8.5 |
| *Galactose :* | |  |  |  |  |
|  | Galactose | -1.6±2 | -67±15.5 | 0.2±12.9 | -45.5±14.7 |
| *Pentose phosphate :* | |  |  |  |  |
|  | Gluconic acid | 116.5±37.1 | -37.4±12.1 | 13.6±7.7 | 35.9±7.5 |
|  | Arabinose | -27.1±29.6 | -3.7±0.2 | 49.4±26.6 | -2.2±23.8 |
| *Glycolysis + neoglucogenesis :* | |  |  |  |  |
|  | Glucose | 3.9±14.2 | 108.6±30.9 | -27±13.2 | -11.4±41.8 |
|  | Lactic acid | 112.2±24.2 | -29.2±7.4 | -8.1±3.1 | -80.7±25.1 |
|  | Galactose-6-phosphate | 996±133.4 | -65.8±5.2 | NA | NA |
| TCA CYCLE | |  |  |  |  |
|  | Isocitric acid | 27.7±8.3 | 4.5±4.6 | 16.1±12.1 | 17.4±2.5 |
|  | Malic acid | -30±12.1 | -28.9±22.8 | 93.5±27 | -58.7±15.2 |
|  | Itaconic acid | 377.9±182,9 | NA | NA | NA |
|  | Aconitic acid, cis- | -627±155 | NA | 871±282 | NA |
|  | Glutaric acid | 313.7±84.9 | -33.7±1 | 29±17 | -5.6±2 |
| AMINO ACIDS METABOLISM | |  |  |  |  |
| *Lysine :* | |  |  |  |  |
|  | Lysine | -93±20.2 | 125.4±64.4 | 10±1.2 | -14.2±5.5 |
| *Alanine, aspartic acid, glutamate :* | |  |  |  |  |
|  | Asparagine | 254.5±33.7 | 9.1±1.2 | 48.5±26.3 | -44.1±23.3 |
| *Glycine, serine, threonine :* | |  |  |  |  |
|  | Glycine | 1. 9±14.1 | -21.8±10 | -45.1±4,6 | -74.1±20.3 |
|  | Homoserine | NA | 16,7±1,6 | -23,6±3.6 | NA |
|  | Threonine, L- | 62.5±0.6 | -65.4±29.1 | 18.8±2.4 | 213.3±64,8 |
| *Aromatic amino acids :* | |  |  |  |  |
|  | Phenylalanine | 114±39,4 | NA | NA | NA |
|  | Shikimic acid | 12.5±26.3 | 78.1±28.8 | 69.8±19.1 | 35.8±3.1 |
| UREA CYCLE METABOLISM | |  |  |  |  |
|  | Ornithine | -35.5±7.8 | NA | 176.1±61.1 | 14.4±3.4 |
| POLYOLS | |  |  |  |  |
|  | Mannitol | 25.8±12.8 | NA | 75.3±33 | NA |
|  | Inositol, myo- | 40.1±31.9 | 35.6±7.9 | -19.7±9.8 | -2.9±1.8 |
|  | Galactitol | NA | 134.9±64.1 | 52.7±35 | -45.6±11.4 |
|  | Maltitol | NA | 97.1±17 | 976,4±109 | NA |
|  | Xylitol | 52,9±34,7 | -22,4±20,5 | 17,3±27,2 | -50,6±26,7 |
| PHENOLIC COMPOUNDS | |  |  |  |  |
|  | Caffeic acid, trans- | NA | -58.2±10.5 | -67.7±6.3 | -40.5±10.8 |
|  | Cinnamic acid, 4-hydroxy-, trans- | -4.1±2.4 | NA | 122.3±20.8 | -17±8.7 |
| PURINE AND PYRIMIDINE METABOLISM | |  |  |  |  |
|  | Guanosine | NA | -50.4±6.1 | 134.4±23.1 | -79.2±47 |
|  | Cytosine | 96.1±40.7 | -71.6±17.4 | 65.6±31.8 | -19.5±4.2 |
| OTHER PATHWAY METABOLISM | |  |  |  |  |
|  | Ethanolamine | NA | -16,7±7,1 | NA | NA |
|  | Phosphoric acid | 16.2±15.3 | -38.9±41.8 | 67.7±44.6 | -58.4±21.3 |
|  | Diethylenglycol | 281.3±51.1 | 32.1±7.4 | -19.4±9.2 | 24.4±14.7 |
|  | Glyceric acid | 42.5±2 | -31.1±2.8 | 63.9±3.2 | -36.6±2 |
|  | Galacturonic acid-1-phosphate | 9.8±1.6 | NA | NA | NA |
|  | Quinic acid, 5-caffeoyl-, trans- | -2.2±7.6 | -64.4±35.5 | -37.5±19.4 | -26.9±2.3 |
|  | Glycinamide | 22.5±128.8 | NA | -2.4±1.6 | -71.3±17.7 |
|  | Malonic acid, methyl- | -74±15.2 | -15.6±0.6 | -45.3±1.6 | -56.5±2 |
|  | Butyric acid, 2-amino- | NA | -12.8±9.7 | -34.2±13.9 | 28.5±3.6 |
|  | Glucopyranose, D- | 59.3±9.1 | -72.3±21.2 | 188.4±66.1 | -52.5±14.5 |
| UNKNOWN COMPOUNDS | |  |  |  |  |
|  | A251001 | -32.9±6.2 | 28±72.3 | -13.3±9.9 | -14.1±44.1 |
|  | A217007 | -31.1±7.3 | -17.5±8.5 | -46.7±14.2 | 649.2±68.5 |
|  | A306003 | 191.5±24.5 | -65.4±15.7 | NA | -41.3±5.3 |
|  | A319008 | 178.8±98.1 | 221.5±11.9 | -44.4±16.4 | -42.9±17.8 |
|  | A285001 | 109±30.1 | -68.2±39,8 | 164.3±46.4 | -68.2±22.3 |
|  | A217004 | NA | -73±42.1 | 37.8±17.5 | 361.6±64.1 |
|  | A229001 | 141±47.1 | -38.1±12.1 | 13.3±4.1 | -47.5±12 |
|  | A260006 | 296.5±85.8 | -55.4±6 | 169.1±58.1 | -79.3±21.7 |
|  | A264005 | 86.7±150.4 | 25.4±126.7 | -7.5±117.8 | -16.8±62.4 |
|  | A199004 | 10.5±3.9 | NA | 373.1±68.7 | -52.3±11.6 |
|  | A170001 | -29±6.5 | 10.9±22 | 2.7±3.8 | 0.8±31.1 |
|  | A155014 | 42±27.8 | 19.5±9.3 | -13.3±22 | 5.4±1.8 |
